# Radiation-Induced Glycogen Accumulation Detected by Single Cell Raman Spectroscopy Is Associated with Radioresistance that Can Be Reversed by Metformin

**DOI:** 10.1371/journal.pone.0135356

**Published:** 2015-08-17

**Authors:** Quinn Matthews, Martin Isabelle, Samantha J. Harder, Julian Smazynski, Wayne Beckham, Alexandre G. Brolo, Andrew Jirasek, Julian J. Lum

**Affiliations:** 1 Trev and Joyce Deeley Research Centre, BC Cancer Agency, Victoria, BC, Canada; 2 Department of Physics and Astronomy, University of Victoria, Victoria, BC, Canada; 3 Department of Biochemistry and Microbiology, University of Victoria, Victoria, BC, Canada; 4 Department of Medical Physics, BC Cancer Agency, Victoria, BC, Canada; 5 Department of Chemistry, University of Victoria, Victoria, BC, Canada; 6 Mathematics, Statistics, Physics, and Computer Science, University of British Columbia Okanagan, Kelowna, BC, Canada; Technische Universitaet Muenchen, GERMANY

## Abstract

Altered cellular metabolism is a hallmark of tumor cells and contributes to a host of properties associated with resistance to radiotherapy. Detection of radiation-induced biochemical changes can reveal unique metabolic pathways affecting radiosensitivity that may serve as attractive therapeutic targets. Using clinically relevant doses of radiation, we performed label-free single cell Raman spectroscopy on a series of human cancer cell lines and detected radiation-induced accumulation of intracellular glycogen. The increase in glycogen post-irradiation was highest in lung (H460) and breast (MCF7) tumor cells compared to prostate (LNCaP) tumor cells. In response to radiation, the appearance of this glycogen signature correlated with radiation resistance. Moreover, the buildup of glycogen was linked to the phosphorylation of GSK-3β, a canonical modulator of cell survival following radiation exposure and a key regulator of glycogen metabolism. When MCF7 cells were irradiated in the presence of the anti-diabetic drug metformin, there was a significant decrease in the amount of radiation-induced glycogen. The suppression of glycogen by metformin following radiation was associated with increased radiosensitivity. In contrast to MCF7 cells, metformin had minimal effects on both the level of glycogen in H460 cells following radiation and radiosensitivity. Our data demonstrate a novel approach of spectral monitoring by Raman spectroscopy to assess changes in the levels of intracellular glycogen as a potential marker and resistance mechanism to radiation therapy.

## Introduction

Tumor cells exhibit altered signaling pathways and metabolic processes that contribute to tumor cell resistance to systemic anti-cancer agents and radiation therapy. One hallmark of tumor cells is the reprogramming of energy metabolism, most commonly described as increased glucose uptake and glycolytic metabolism. This inherent metabolic property of cancer cells has been suggested to alter the sensitivity to radiation [1–5]. Many of these pathways are under investigation as candidate molecular targets to sensitize tumor cells to cell death when combined with radiation therapy. However, the success of this approach will require assessment and early monitoring of tumor cells that can identify metabolic features capable of conferring radiation sensitivity.

Raman spectroscopy can provide label-free molecular information from single live cells. Raman spectroscopy has been applied to discriminate between various cell types, both healthy [6] and pathological [7, 8]. Moreover, Raman spectroscopy is able to monitor molecular and metabolic changes within a given cell population. Recent work with single cell Raman spectroscopic techniques have proven sensitive to detect metabolic changes due to the differentiation of human embryonic stem cells into lineage specific cardiac cells, where the dominant Raman feature responsible for discrimination was found to be intracellular glycogen content [9, 10]. Raman spectroscopy is highly sensitive to detect and quantify variability in absolute intracellular glycogen content [11]. Thus, intracellular glycogen observed with Raman spectroscopy may serve as a key bio-response marker during different cellular processes.

Glycogen is a polymer of glucose residues linked together by α-(1,4)-glycosidic bonds and is found primarily in the liver. During the fed state, an increase in glucose levels stimulates insulin-mediated activation of glycogen synthase, the primary enzyme involved in joining monomers of UDP-glucose to form glycogen. In the fasted state, glycogen is broken down by glycogen phosphorylase into monomers of glucose-1-phosphate. Intracellular glycogen can be detected in different tumor cells [12–14] and may provide metabolic precursors to protect against hypoxia and other forms of stress [15–17]. Glycogen metabolism is regulated by a diverse set of signaling pathways that are involved in tumor progression. In particular, the catalytic activity of glycogen synthase is directly modulated through phosphorylation by glycogen synthase kinase (GSK-3), AMP-activated kinase (AMPK), protein kinase A and casein kinase 2. Thus, phosphorylation of glycogen synthase leads to its inactivation and a reduction in the capacity to synthesize glycogen. While a direct role for glycogen in radiation sensitivity has not been reported, both GSK-3 and AMPK have been implicated in the cellular responses to radiation [2].

Single cell Raman spectroscopy used in conjunction with principal component analysis (PCA) is sensitive to molecular and metabolic changes within human cancer cells responding to clinically relevant single low and high doses of ionizing radiation [18, 19]. The radiation responses observed with Raman spectroscopy are cell-line dependent and segregate according to both p53 status and intrinsic radiosensitivity, but not tissue of origin [20]. In this study, we report that the dominant radiation response observed in radioresistant tumor cell lines arises from a radiation-induced metabolic switch that can be detected by Raman spectroscopy as an increase in intracellular glycogen abundance. This accumulation of glycogen coincided with an increase in GSK-3β and AMPKα signaling, and was associated with radiation resistance. In radioresistant MCF7 breast tumor cell lines, co-treatment with metformin resulted in enhanced cell death and loss in viability. This resulted in a significant reduction in radiation-induced glycogen accumulation and increased radiosensitivity. Thus, our data show that Raman spectroscopic detection of glycogen levels provides a powerful approach to assess metabolic changes that could render tumor cells resistant to radiation therapy.

## Materials & Methods

### Cell lines

H460 (ATCC# HTB-177), MCF7 (ATCC# HTB-22) and LNCaP (ATCC# CRL-1740) cells were obtained as original stocks with a Certificate of Analysis from American Type Culture Collection (ATCC, Manassas, VA, USA). Cells were cultured as monolayers, at 37°C and 5% CO_2_, and were subcultured every 3–4 days to maintain exponential growth. Cells were cultured in RPMI 1640 (H460 and LNCaP) or DMEM (MCF7) and as previously described [19–21]. All media components were purchased from Hyclone.

### Irradiation and metformin treatment

Cells were harvested and equivalent aliquots were incubated for 4 days at an initial cell density determined to achieve 50% confluency at time of irradiation. One hour prior to irradiation, culture media was replaced with either fresh media or media containing 5 mM metformin (Sigma Aldrich Canada Co., Oakville, Canada). Cell monolayers were irradiated with a single fraction of 6 MV photons from a Varian 21EX linear accelerator (Varian Medical Systems, Palo Alto, CA, USA) at a dose rate 6 Gy/minute. Single fractions of 0, 2, 4, 6, 8, and 10 Gy were delivered to 3 cultures per dose.

### Cell cycle and viability analysis

Cell cycle distribution of each culture was determined with propidium iodide flow cytometry, as described previously [21]. A portion of the cell suspension was used to determine both absolute cell numbers and the fractions of viable and dead cells using the Guava ViaCount assay (EMD Millipore, Etobicoke, Ontario, Canada). Statistical analysis was performed using GraphPad Prism version 6.0 (GraphPad Software, La Jolla, CA,USA).

### Clonogenic survival assay

For each sample harvested for Raman spectroscopic analysis on day 1 post-irradiation, suspensions were serially diluted, counted and seeded in triplicate into 100-mm dishes at cell numbers pre-determined for each dose and cell line. Cultures were incubated at 37°C for 7–14 days (depending on cell line). Cells were washed with PBS, fixed with 10% formalin for 30 minutes, rinsed in PBS, and stained with 0.05% crystal violet for 30 minutes. The dishes were rinsed and air dried overnight. Colonies exceeding 50 cells were counted by analyzing high-resolution images of the dishes using an in-house Matlab (Mathworks Inc., Natick, MA, USA) program. Plating efficiencies and surviving fractions were determined for each experiment following established protocols [22] and repeated in triplicate. Curve fitting and statistical analysis was performed using GraphPad Prism.

### Western blot analysis

Cell lysate preparation and western blotting was performed as previously described [23]. Proteins were probed with primary antibodies against p-GSK-3β (Ser9) (rabbit monoclonal, clone 5558, Ab Registry# AB_10695601), GSK-3β (rabbit monoclonal, clone 9315, Ab Registry# AB_10694680), p-AMPKα (Thr172) (rabbit monoclonal, clone 2535, Ab Registry# AB_331250), AMPKα (rabbit polyclonal, clone 2532, Ab Registry# AB_10694064) and p21 (rabbit monoclonal, clone 2947, Ab Registry# AB_823586) (all 1:1000 dilution from Cell Signaling Technology, Cell Signaling Technology, Danvers, MA, USA). Actin primary antibodies were used as a loading control (mouse monoclonal, clone AC-15, catalog# A5441, Sigma Aldrich), prepared at a 1:50000 dilution. Antibodies against p53 (mouse monoclonal, Ab Registry #AB_628082) were obtained from Santa Cruz Biotechnology (Santa Cruz, CA, USA) and were used at 1:250 dilution. Membranes were washed three times in TBST and hybridized using secondary antibodies Alexa Fluor 680-conjugated donkey anti-mouse ([Ea] Ass, polyclonal, catalog# A10038, Life Technologies Inc., Burlington, Canada) or IRDye 800-conjugated goat anti-rabbit (Rabbit, polyclonal, catalog# 605-432-003, Rockland Immunochemicals Inc., Limerick, PA, USA), both at 1:10000 dilution in 5% (w/v) milk in TBST for 1 hour. Membranes were washed three times with TBST and protein bands were imaged on an Odyssey infrared imaging system (Li-Cor Biosciences, Santa Clara, CA, USA). Densitometry was performed in Matlab using in-house software for S3 Fig. We performed three independent experiments for western blots presented in this manuscript. Each figure was generated as a composite from the same gel with non-adjacent bands separated by a vertical line.

### Raman spectroscopic acquisition and spectral processing

Cell preparation for Raman spectra acquisition, and processing was performed as described previously [21]. Briefly, cells were washed with PBS, harvested with trypsin and centrifuged into a pellet. Pellets were transferred to a 5 mm thick magnesium fluoride window (Janos Technology Inc., Keene, NH, USA) and allowed to air dry for 5 minutes before spectral acquisition. Raman spectra were acquired from 20 individual cells from each sample (20 spectra per sample at the radiation doses indicated) over 3 days of analysis performed in triplicate for all 3 cell lines. Cells for analysis were chosen at random from the top layer of the cell pellet. Spectral acquisition was performed with an inVia Raman microscope (Renishaw Inc., Gloucestershire, UK) with a 100X dry objective (NA = 0.9) (Leica Microsystems, Concord, Ontario, Canada), a 600 lines / mm diffraction grating, a 10 second acquisition time per cell and a 450–1800 cm^-1^ spectral window. Spectra were recorded with a thermoelectrically cooled iDus CCD detector (Andor Technology, Belfast, UK). A 785 nm laser (Renishaw) was used for excitation. The laser power density at the sample was 0.5 mW / μm^3^ with a sampling volume of 2 x 5 x 10 μm to allow single cell Raman spectrum acquisition. Each cell spectrum was processed to remove cosmic rays, correct for wavenumber calibration drifts, estimate and subtract a baseline arising from the substrate and biological fluorescence, and normalize to the total amount of biological material within the sampling volume. The fully processed data sets were analyzed with principal component analysis using standard algorithms in Matlab. Statistical analysis of PCA score distributions was performed using GraphPad Prism.

### Intracellular glycogen assay

Cellular glycogen levels were assessed using the glycogen assay kit (BioVision Inc., Milpitas, CA, USA). Briefly, one million cells were harvested into suspension, lysed and boiled for 10 min at 100°C. Lysates were spun down at 14,000 rpm for 10 min. The supernatant was collected and protein concentration was determined for each sample. Samples were prepared in parallel to measure the glucose background, by removal of the hydrolysis enzyme step. Samples for each radiation dose were plated in triplicate on a 96-well plate.

## Results

### Raman spectroscopy detects radiation-induced glycogen accumulation in radioresistant H460 and MCF7 cells, but not in radiosensitive LNCaP cells

**Fig 1:**
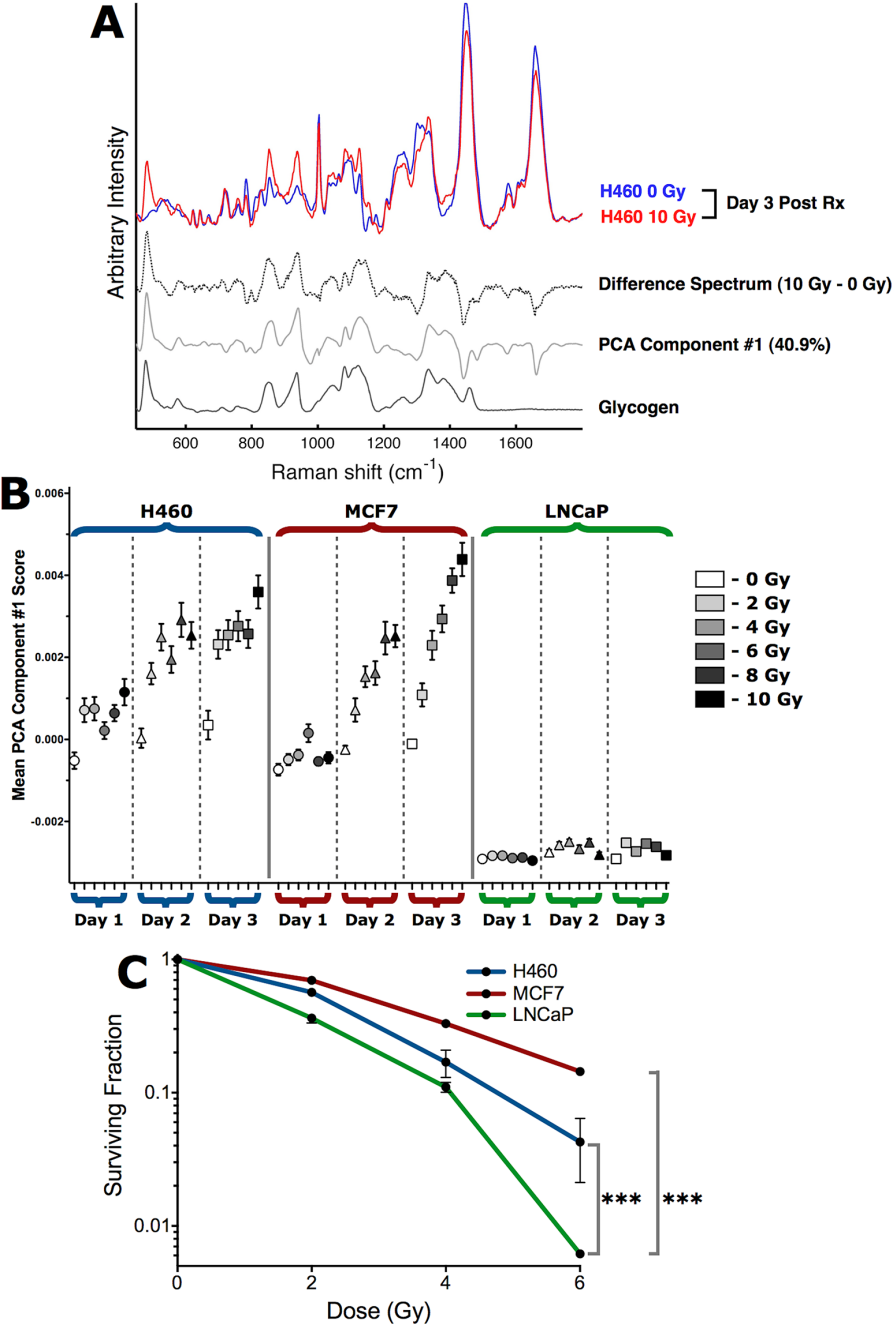
Single-cell Raman spectroscopy with PCA detects early radiation-induced glycogen synthesis in H460 and MCF7 cells, but not in radiosensitive LNCaP cells. (A) Single-cell Raman spectra of an irradiated H460 cell (10 Gy) and an unirradiated H460 cell at 3 days post-irradiation demonstrate the Raman spectroscopic detection of increased intracellular glycogen in irradiated H460 and MCF7 cells. The difference spectrum (dashed trace) is shown for comparison with the first PCA component (solid gray trace) from the entire Raman spectroscopy data set presented in B. The Raman spectrum of glycogen (black trace) is shown for comparison. (B) H460, MCF7 and LNCaP cells were irradiated with single fractions of radiation, and single-cell Raman spectra were collected from 20 cells from each sample post-irradiation. Experiments were performed in triplicate, resulting in 60 single-cell spectra per sample condition, and 3240 spectra overall. The mean PCA scores (n = 60 spectra per point, error bars are ± SE) for the first PCA component indicate statistically significant (p<0.05 by unpaired two-tailed t-test) increases in intracellular glycogen, relative to same day unirradiated cells. (C) Clonogenic survival of irradiated H460, MCF7 and LNCaP cells. Data are the mean ± SE from three independent experiments, each cultured in triplicate. *** p<0.001 (extra sum-of-squares F test).

In the current work, we asked whether Raman spectroscopy could identify spectral features that change post-radiation and if those changes would be different for radiosensitive and radioresistant human tumor cell lines. Two radioresistant human tumor cell lines were selected, a non-small cell lung tumor cell line, H460, and the estrogen receptor positive breast cancer cell line, MCF7. The prostate tumor cell line, LNCaP, was chosen as a radiosensitive control cell line. Each cell line was cultured to 50% confluency and subjected to clinically relevant single fractions of 2–10 Gy radiation. On day 1, 2, and 3 post-irradiation, single-cell Raman spectra were acquired as previously described [18, 20]. As shown in Fig 1A, we observed marked differences in Raman features for H460 cells after radiation treatment. A representative point-by-point difference spectrum and the PCA component from the entire data set are both dominated by Raman spectral features of glycogen. A similar change in this glycogen signature was found in irradiated MCF7 cells, but not in the radiosensitive control LNCaP cells. The complete Raman data set in Fig 1A and 1B comprised 3240 single-cell spectra (see Materials and Methods). The first PCA component accounts for 40.9% of the total variance, and represents the dominant observation of variability in intracellular glycogen content within all cells in the complete Raman data set. The mean PCA scores for the first PCA component (Fig 1B) indicate that statistically significant (p<0.05) increases in intracellular glycogen, relative to same day un-irradiated cells, occurred for all radiation doses at days 1–3 for H460 cells and at days 2–3 for MCF7 cells. In LNCaP cells, we did not detect any significant change in glycogen levels regardless of dose or time.

To investigate possible biological endpoints correlating with this radiation-induced glycogen change, we performed proliferation, viability, cell-cycle, and clonogenic assays in parallel with the Raman analysis. On day 3 post-irradiation and for each dose that was tested, all three tumor lines exhibited statistically consistent reductions in proliferation and increases in cell death with a dose-dependent trend in both of these endpoints (S1 and S2 Figs). The decrease in cell proliferation was consistent with cell cycle redistribution in response to 2–6 Gy radiation, with a common trend towards accumulation of cells in the G_2_-fraction at the expense of G_1_- and S-phase fractions (S2 Fig). Interestingly, despite these comparable responses to radiation the clonogenic survival assays indicated that both H460 and MCF7 cells are significantly more radioresistant than LNCaP cells (Fig 1C). As such, it is possible that glycogen, or the radiation-induced signaling pathways that promote glycogen accumulation, could contribute to the observed resistance to radiation in H460 and MCF7 cells.

### The cell cycle regulators p53 and p21 do not correlate with changes in glycogen following radiation

To examine whether it was possible to ascribe changes in glycogen to the deregulation of p53 and p21, two critical cell cycle checkpoints modulators, we assessed p53 and p21 levels post-irradiation. All three cell lines, H460, MCF7 and LNCaP, express wild-type p53 [24–26], and exhibited higher expression of both p53 and p21 in response to radiation (S3 Fig). Thus, the up-regulation of p53 and p21 did not correlate with the observed radiation-induced glycogen synthesis or increased radioresistance in H460 and MCF7 cells.

### Radiation-induced glycogen accumulation in H460 and MCF7 cells correlates with changes in GSK-3β, but not AMPK signaling

**Fig 2:**
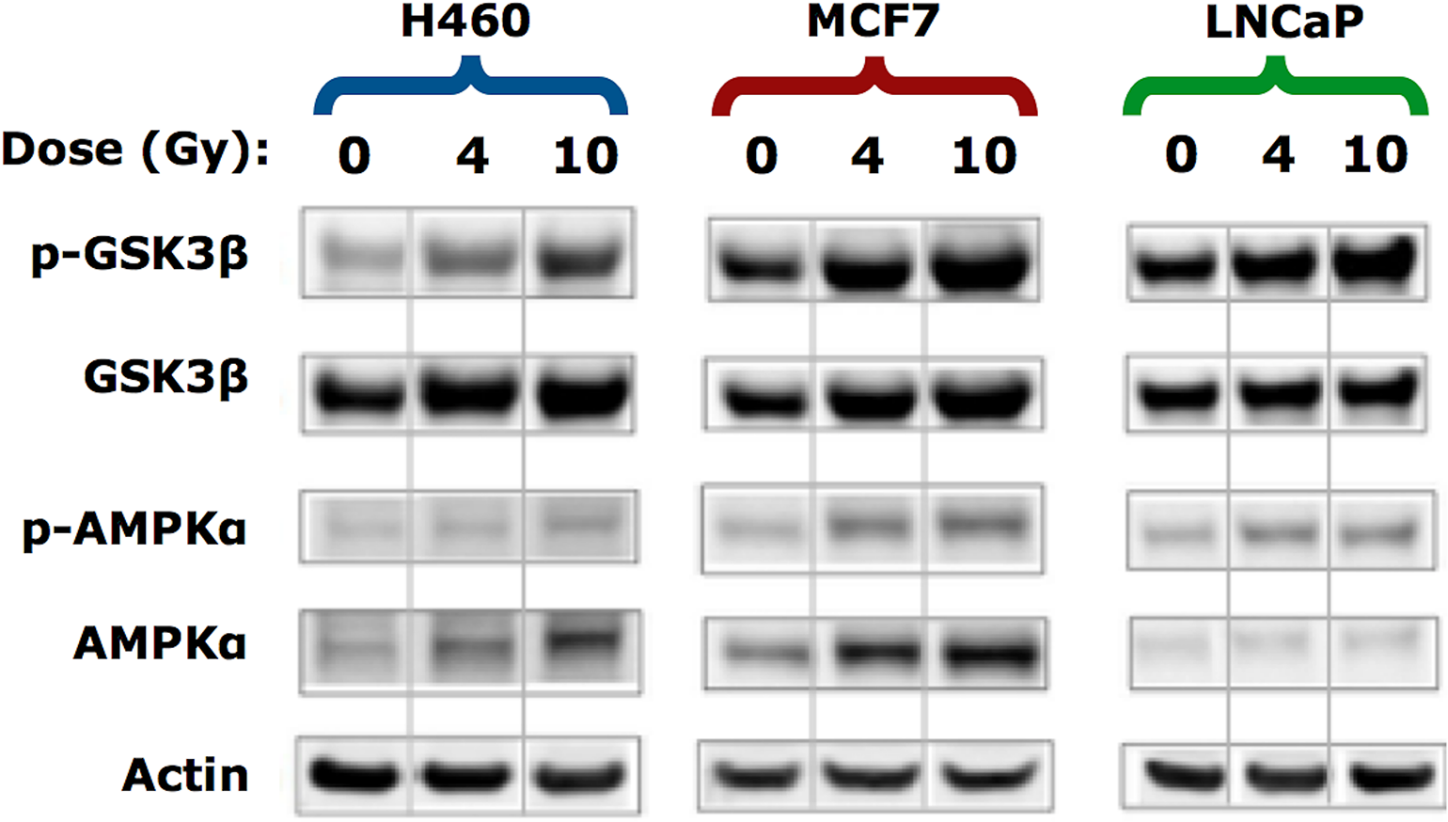
Western blotting for the effect of irradiation on signals associated with the regulation of glycogen synthesis and utilization. Whole cell lysates were prepared 3 days post-irradiation and Western blot analysis was conducted using the indicated antibodies. Blots are representative of three experiments. Actin was used as a loading control. Each panel was generated as a composite from the same gel with non-adjacent bands separated by a vertical line.

To investigate the possible signaling pathways involved in radiation-induced glycogen accumulation, we examined the expression of glycogen synthase kinase 3β (GSK-3β) and AMP-activated protein kinase alpha (AMPKα), two important kinases that have been implicated in cancer cell survival and radiation response. The GSK-3 serine-threonine kinase has two major isoforms. In particular, GSK-3β is involved in energy metabolism and has been implicated in radiation cytotoxicity responses [4] by protecting cells from apoptosis [27]. The phosphorylation of GSK-3β on serine 9 reduces its activity thereby relieving its negative regulation of glycogen synthase. In contrast to un-irradiated controls, we found a dose-dependent increase in phosphorylation of GSK-3β in all three of the tumor lines examined (Fig 2). We also found a small increase in total GSK-3β between 0 Gy and 4 Gy for both H460 and MCF7 cells and to a lesser degree between 4 Gy—10 Gy. Since the amount of total protein as measured by actin was similar in each condition, this suggests that radiation may have both post-translational and transcriptional effects on GSK-3β. Taken together, the radiation-induced accumulation of glycogen could be in part due to inactivation of the GSK-3β pathway and a higher capacity of cells to produce glycogen.

Another key pathway that regulates glycogen synthesis is the AMP-activated protein kinase (AMPKα) [28]. AMPKα activation has been linked to radiation and is known to regulate radiosensitivity [1, 5, 29, 30]. We anticipated that radiation would trigger down-regulation of phosphorylated AMPKα (p-AMPKα) and activate glycogen synthase. However, we found that all irradiated cell lines displayed a small but detectable increase in p-AMPKα (Fig 2). This was unexpected because higher levels of p-AMPKα would be predicted to reduce glycogen synthase activity and lower the capacity to synthesize glycogen. As observed with total GSK-3β, radiation caused an up-regulation in the expression of total AMPK in all three tumor cell lines. Thus, radiation may also have transcriptional and post-translational effects on AMPK. Nevertheless, changes in the levels of AMPK induced by radiation cannot solely account for the accumulation of glycogen that we detected using Raman spectroscopy.

### Raman spectroscopy detects suppression of radiation-induced glycogen accumulation in metformin treated MCF7 cells, but not H460 cells.

**Fig 3:**
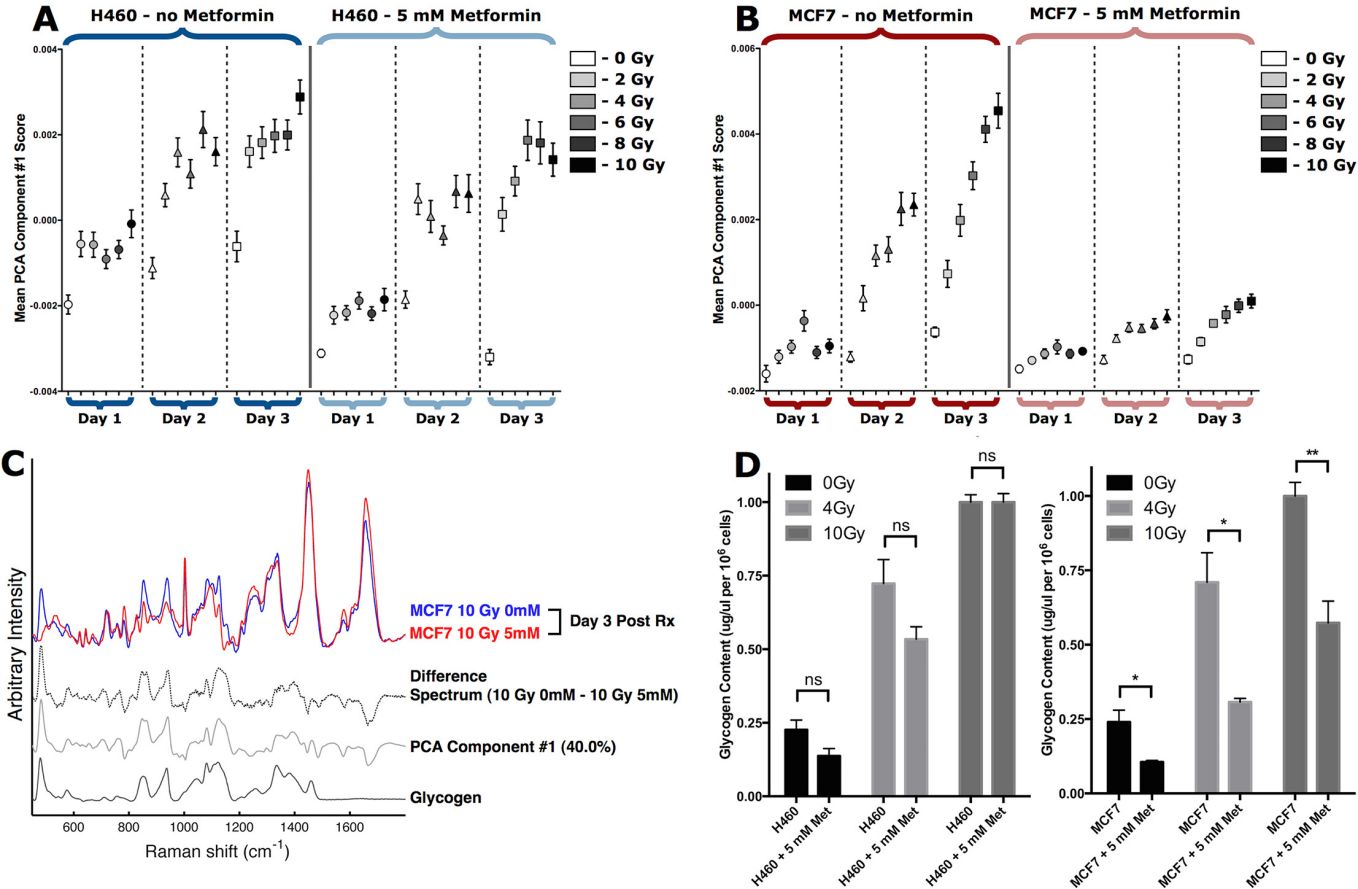
Single-cell Raman spectroscopy detects early and sustained inhibition of radiation-induced glycogen accumulation in metformin treated MCF7 cells. (A, B) H460 and MCF7 cells were treated with or without 5 mM metformin from 1 hour prior to irradiation with single fractions at the doses shown. Single-cell Raman spectra were collected for 20 cells from each sample at 1, 2 and 3 days post-irradiation. Experiments were performed in triplicate, resulting in 60 single-cell spectra per sample condition, and 2160 spectra overall per data set. The mean PCA scores (n = 60 spectra per point, error bars are ± SE) for the first PCA component are shown. (C) Single-cell Raman spectra of 10 Gy irradiated MCF7 cells at 3 days post-irradiation, with and without 5 mM metformin. The difference spectrum (dashed trace) is shown for comparison with the first PCA component (solid gray trace) from the entire Raman spectroscopy data set presented in B. The Raman spectrum of glycogen (black trace) is shown for comparison. (D) Intracellular glycogen levels measured enzymatically 3 days post-irradiation in the presence or absence of 5 mM metformin. Each bar represents the average of 3 independent experiments ± S.D where each sample is assayed in triplicate. Statistical significance was determined using unpaired two-tailed t-test, n.s. not significant, *p<0.05, ** p<0.01.

Metformin is an oral anti-diabetic drug that has been widely used as an activator of AMPKα. The target of metformin remains largely unknown, however, metformin can sensitize a number of tumor cells to radiation [5, 29, 31–35]. In particular, metformin was able to enhance radiosensitivity of MCF7 tumor cells via modulation of AMPKα [30]. To further investigate the relationship between radiosensitivity and the radiation-induced increase in intracellular glycogen observed with Raman spectroscopy, H460 and MCF7 cells were irradiated in the presence or absence of metformin. As shown in Fig 3A, there was minimal effect on radiation-induced glycogen accumulation in H460 cells after incubation with 5 mM metformin for 1–3 days post-irradiation. In contrast, Raman spectroscopy was able to detect a dramatic reduction in glycogen levels in MCF7 cells treated with metformin and radiation (Fig 3B and 3C). The reductions in glycogen levels were significant (p<0.01) for all irradiated MCF7 cultures from 2–3 days. The change in glycogen detected by Raman spectroscopy could be observed in the first PCA component, which accounts for 40.0% of the total variance in the complete Raman data set of 2160 single-cell spectra (Fig 3C).

To corroborate the spectral detection of radiation-induced changes in glycogen, we used a biochemical enzyme assay to assess total intracellular glycogen levels 3 days post-irradiation, with or without metformin. As expected in H460 cells, 10 Gy radiation caused a reproducible 4.5-fold increase in total intracellular glycogen levels. Similarly, MCF7 cells showed a 4.1-fold higher amount of glycogen after radiation (Fig 3D). As we predicted based on the Raman spectra, H460 cells showed no significant change in the amount of glycogen when radiation was given in the presence of 5 mM metformin. In contrast to H460 cells, treatment of MCF7 cells with 5 mM metformin reduced the total amount of glycogen by 40%, from 0.95 μg/uL per 10^6^ cells to 0.55 μg/μL per 10^6^ cells when co-treated with 10 Gy radiation. At the 4 Gy radiation dose, treatment with metformin resulted in more than a 60% reduction in glycogen.

### Metformin mediates suppression of glycogen synthesis signaling pathways

**Fig 4:**
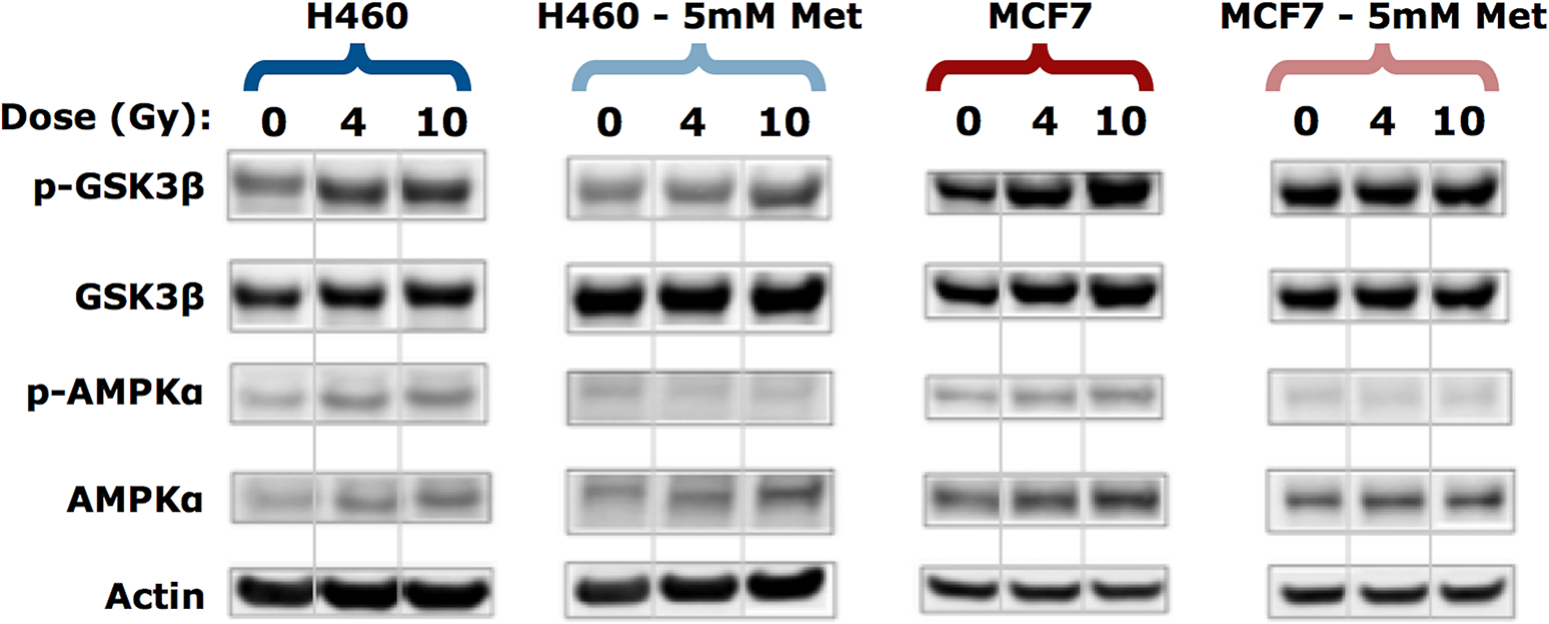
Metformin suppresses GSK-3β but not AMPKα. Western blotting was performed on lysates collected 3 days post-irradiation using the indicated antibodies, n = 3 experiments. Actin was used as a loading control. Each panel was generated as a composite from the same gel with non-adjacent bands separated by a vertical line.

We next examined the effects of radiation plus metformin treatment on glycogen signaling pathways. We analyzed phosphorylation patterns in whole cell lysates prepared on day 3 post-irradiation by Western blot. Both H460 and MCF7 cells treated with 5 mM metformin in the absence of radiation showed no significant change in the level of p-GSK-3β (Fig 4). When H460 cells were treated with radiation and metformin, we observed a reduction in p-GSK-3β and to a lesser extent in MCF7 cells. Thus, metformin at the concentrations used here can reduce p-GSK-3β levels [36].

Despite a small but detectable increase in p-AMPKα levels in H460 and MCF7 cells exposed to increasing doses of radiation, co-treatment with metformin was able to reduce the increase in p-AMPKα (Fig 4). The effects of metformin on p-AMPKα were expected, however, loss of p-AMPKα would generally be consistent with activation of glycogen synthase and higher levels of intracellular glycogen. Nevertheless, the Raman spectra as well as the biochemical glycogen assay indicated that there was a dramatic reduction in radiation-induced glycogen accumulation when MCF7 cells were treated with metformin (Fig 3).

### Metformin-mediated suppression of radiation-induced glycogen accumulation sensitizes cells to cell death

**Fig 5:**
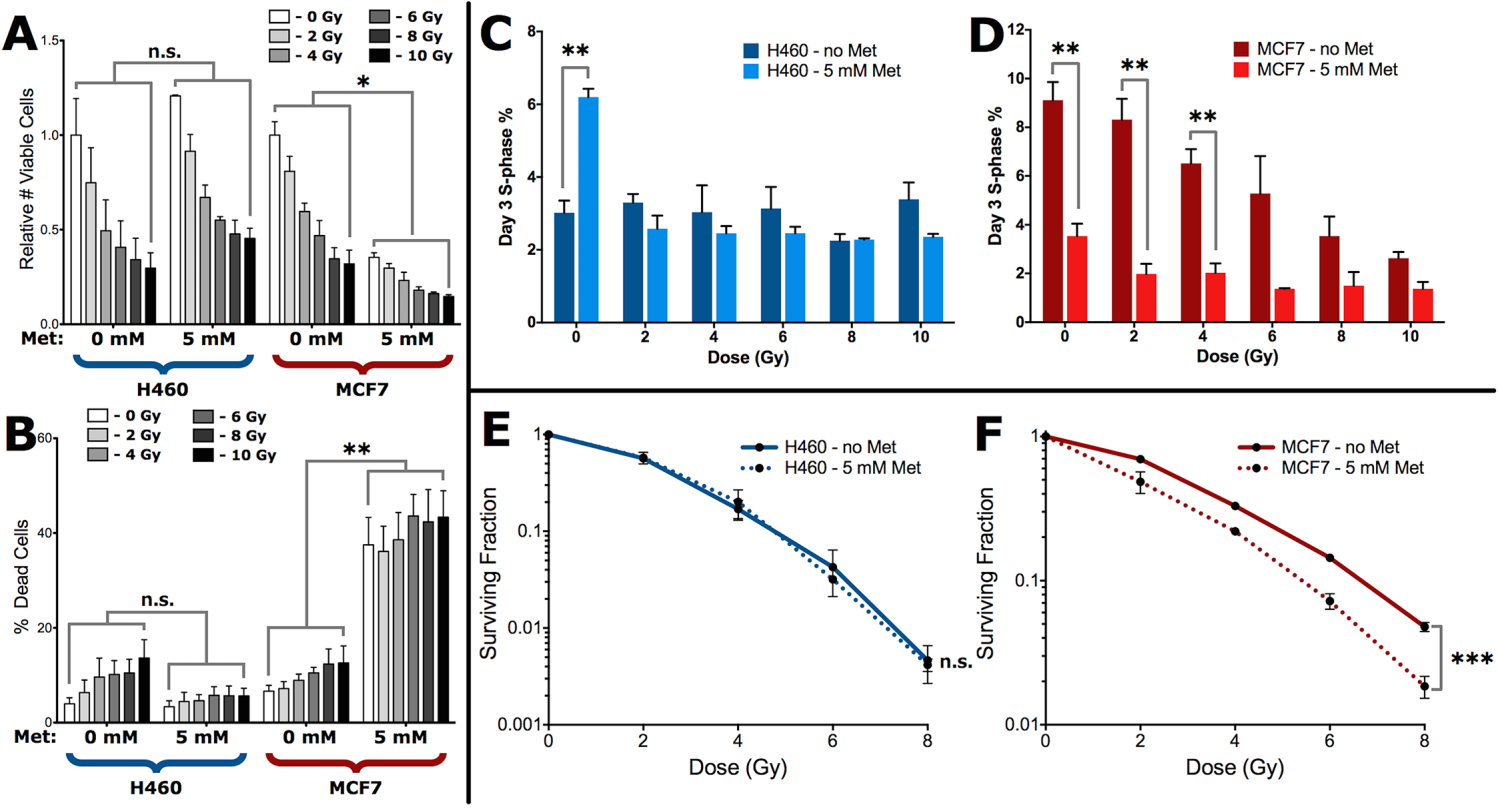
Preferential sensitization of MCF7 cells treated with radiation and metformin. (A) Total numbers of live and (B) dead cells in metformin co-treated H460 and MCF7 cultures were counted in triplicate at 3-days post-irradiation. Viable cell counts in (A) are relative to the untreated control for each cell line. Statistical significance tests shown apply to each dose-matched sample pairing within each group. Cell cycle distributions from each culture were determined via propidium iodide staining, and S-phase fractions for metformin co-treated (C) H460 and (D) MCF7 cells are shown at 3-days post-irradiation. Data are the mean ± SE from 3 independent experiments. * p<0.05, ** p<0.01, and n.s. not significant (unpaired two-tailed t-test). Clonogenic survival of metformin co-treated (E) H460 and (F) MCF7 cells performed 1 day post-treatment as described in the Materials and Methods. Data are the mean ± SE from three independent experiments, each cultured in triplicate. n.s.—no significant difference between curves, *** p<0.001 (extra sum-of-squares F test).

Since metformin was able to reverse the effects of radiation on the accumulation of glycogen, we investigated how this would affect survival despite the effects of metformin on reducing the levels of p-AMPKα. At all the clinically relevant radiation doses tested, there was a dramatic loss in MCF7 cell viability with the addition of 5 mM metformin (Fig 5A). Incubation of metformin also caused the percentage of dead cells to increase from 12.6% to 43.3% after 3 days of co-treatment with 10 Gy of radiation (Fig 5B). In H460 cells, radiation did not change the viability or degree of cell death with metformin treatment (Fig 5A and 5B). We further analyzed changes in the cell cycle profiles following incubation with metformin. Metformin treatment of H460 cells in the absence of radiation resulted in a 2-fold increase in the fraction of cells in S-phase whereas no concomitant differences were observed in G_1_/G_0_ or G_2_ regardless of whether cells were irradiated (Fig 5C, S4A and S4C Fig). In contrast, MCF7 cells showed a 3-fold reduction in S-phase cells with metformin alone (Fig 5D). There was an additional but small increase in the loss of S-phase cells, up to a 4-fold reduction when cells were co-treated with metformin and low doses of radiation (Fig 5D). This decrease in S-phase cells resulted in a concomitant and significant increase in the fraction of G_1_/G_0_ cells, but not G_2_ cells (S4B and S4D Fig). When we investigated this combination treatment using radiosensitivity assays, irradiated H460 cells had similar levels of clonogenic survival in cells treated with (SF2 = 0.58) or without metformin (SF2 = 0.57) (Fig 5E). In contrast, the addition of metformin to irradiated MCF7 cells resulted in significant loss in clonogenic survival at all doses of radiation (Fig 5F).

## Discussion

There remains an unmet need for new approaches that can predict or monitor patient responses to radiation, and identify patients that may benefit from combination therapies. Label-free Raman spectroscopy coupled with chemometrics, such as PCA, is a potential way to discriminate between cell types, and to monitor biochemical changes within a given population of cells. This study has shown its application in detecting radiation-induced changes in glycogen that are associated with radiosensitivity. A key feature of our observations is the detection of changes in glycogen following radiation treatment in two radioresistant cell lines MCF7 and H460 but not in radiosensitive LNCaP cells. More importantly, Raman spectroscopic changes can be assessed with varying clinical doses of radiation, and when combined with the drug metformin, could lead to enhancements in radiotherapy. Thus, single-cell Raman spectroscopy has the potential to detect and distinguish novel biochemical signatures that are associated with tumor cell radioresistance.

Consistent with previous reports showing that inactivation of GSK-3β can protect against a wide variety of normal cell types from cell death and radiation injury [37, 38], we found that radiation treatment led to a greater inactivation of GSK-3β. The role of GSK-3β in radiation cytotoxic response in tumor cells is complex due to its multifunctional regulation of other key cellular pathways including cell proliferation, survival, cell cycle and cell differentiation. There is also conflicting evidence suggesting that blocking GSK-3β can either sensitize or promote resistance of pancreatic tumor cells to chemoradiation [4, 39]. This further highlights that other factors and pathways may work together to determine radiation responses and are likely dependent on the oncogenic characteristics inherent to a given tumor cell line or type of tumor.

The liver kinase B1 (LKB1) and AMPKα/mTOR pathways play a central role in tumor cell survival in response to cellular stress. Activation of AMPKα can also target glycogen synthase and reduce the capacity of glycogen synthase to generate glycogen. We found that there was a small change in p-AMPKα for H460 cells but a more pronounced increase was observed in MCF7 following radiation. This might be explained by the expression of mutant LKB1 in H460 cells [40]. This is in agreement with other studies showing that the expression of both total AMPK and activated p-AMPKα increases after 8 Gy irradiation and this led to a radioresistant phenotype [1, 29]. It is possible that other signaling pathways that control glycogen metabolism including PP1 and PYGL may also explain the higher levels of radiation-induced glycogen observed here. Moreover, the pattern of phosphorylation on glycogen synthase might be crucial for its activity. Therefore, we cannot rule out the possibility that other phosphorylation or dephosphorylation events are responsible for the change in glycogen levels following radiation. However, based on our results it is reasonable to speculate that the increase in p-GSK-3β following radiation increases the capacity of glycogen synthase to produce and accumulate intracellular stores of glycogen. Indeed, our observations correlate with higher levels of radiation-induced glycogen accumulation for H460 and MCF7 but not LNCaP as detected by Raman spectroscopy.

Several studies show that metformin and phenformin have synergistic effects with ionizing radiation on inducing tumor cell death [30, 31, 35, 41]. In contrast to H460 cells, we observed a significant inhibition of glycogen accumulation in MCF7 cells treated with metformin, which may be due to the loss of function of mutated LKB1 in the H460 cell line. The first PCA component showed that glycogen represented approximately 40% of the total variance in each data set, indicating that Raman spectroscopy can reliably detect differences in glycogen content in irradiated cells treated with or without metformin. In our study and in agreement with previous work, radiation alone led to an increase in p-AMPKα [30]. We expected the combination of metformin and radiation might further increase p-AMPKα expression because higher levels of p-AMPKα would inactivate glycogen synthase and thus reduce the capacity to synthesize glycogen. Our Raman dataset and biochemical assay for intracellular glycogen showed that metformin treatment resulted in a reduction in radiation-induced glycogen accumulation in MCF7 cells. However, we found that irradiation and co-treatment with metformin caused a significant decrease in p-AMPKα levels, a finding that differs from the purported role of AMPK regulation of glycogen synthesis as well as a recent study by Song and colleagues [30]. One possible explanation is that we treated cells with metformin and radiation at the same time, while in the study by Song *et al*., [30] cells were treated with metformin for up to 48 hours prior to exposure to radiation. Another possibility is that radiosensitization by metformin is independent of AMPK [42, 43]. Because changes in signaling pathways in response to radiation are transient, it could be possible that other signaling pathways such as GSK-3 work in concert with AMPK to fine tune the production and degradation of glycogen. Our findings also raise the possibility that monitoring signal transduction cascades could lead to misinterpretation of biological responses to radiation because many of these pathways are mutated or redundant in tumors cells. Such assessment of signaling events could also be complicated by the multiple downstream targets that each pathway can regulate. Therefore, one novel approach, as demonstrated by our study, is to use label-free Raman spectroscopy to directly assess biochemical changes that are associated with radiation responses.

Despite some of the biochemical differences in the signaling events that occur with metformin treatment, our data, to the best of our knowledge, is the first report of Raman detection of radiation-induced glycogen accumulation in tumor cell lines. Although it is still unclear how metformin exerts its anti-cancer properties, monitoring glycogen and other metabolic changes post-irradiation by Raman spectroscopy provides a new approach to assist in developing personalized treatments that have the highest likelihood of improving responses to radiation treatment.

## Supporting Information

S1 Fig H460, MCF7 and LNCaP cells exhibit statistically consistent reductions in proliferation and increases in death for each dose at day 3 post-irradiation. Total numbers of (**a**) live and (**b**) dead cells in irradiated cultures were counted in triplicate at 3-days post-irradiation. Viable cell counts in (**a**) are relative to the untreated control for each cell line. Values are the mean ±SE from 3 independent experiments.
(TIFF)

S2 Fig Cell cycle distributions of H460, MCF7 and LNCaP cells at day 3 post-irradiation. Relative fractions of (**a**) G1/G0-, (**b**) S-, and (**c**) G2-phase cells were counted in triplicate at 3-days post-irradiation via propidium iodide flow cytometry. Values are the mean ±SE from 3 independent experiments.
(TIFF)

S3 Fig Quantification of Western blots for the effect of 2–10 Gy irradiation on (a) p53 and (b) p21 expression in H460, MCF7 and LNCaP cells. Whole cell lysates were prepared 3 days post-irradiation and Western blot analysis was conducted using anti–p53,–p21, and–actin antibodies. Results are the mean ±SE from two independent experiments.
(TIFF)

S4 Fig Cell cycle distributions for radiation and metformin co-treated H460 and MCF7 cells determined via propidium iodide flow cytometry. (**a** & **b**) G1/G0-phase fractions for (**a**) H460 and (**b**) MCF7 cells, and (**c** & **d**) G2-phase fractions for (**c**) H460 and (**d**) MCF7 cells were measured in triplicate at 3-days post-irradiation. Values are the mean ±SE from 3 independent experiments. ** p < 0.01 (unpaired two-tailed t-test).
(TIFF)
