# Supplementary figures and images for: Radiation-Induced Glycogen Accumulation Detected by Single Cell Raman Spectroscopy Is Associated with Radioresistance that Can Be Reversed by Metformin
Source: PLoS One. 2015 Aug 17;10(8):e0135356. doi: 10.1371/journal.pone.0135356 (PMC4539228; doi:10.1371/journal.pone.0135356)

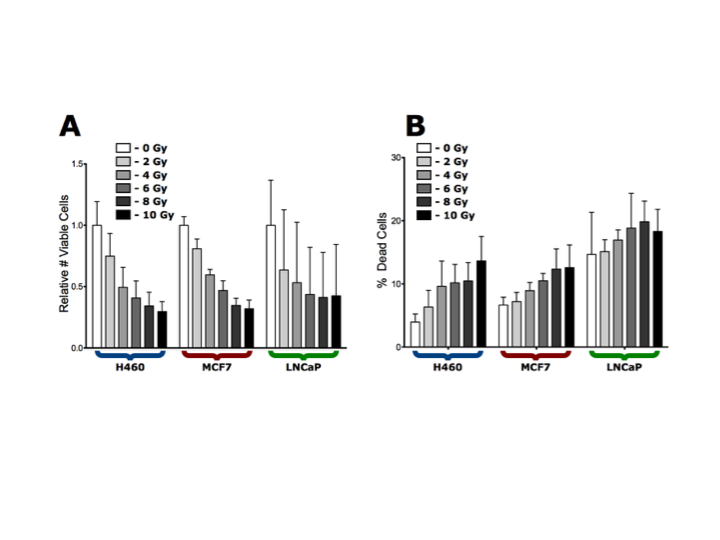

Supplement: S1 Fig — Total numbers of (a) live and (b) dead cells in irradiated cultures were counted in triplicate at 3-days post-irradiation. Viable cell counts in (a) are relative to the untreated control for each cell line. Values are the mean ±SE from 3 independent experiments. (TIFF) [file pone.0135356.s001.tiff]

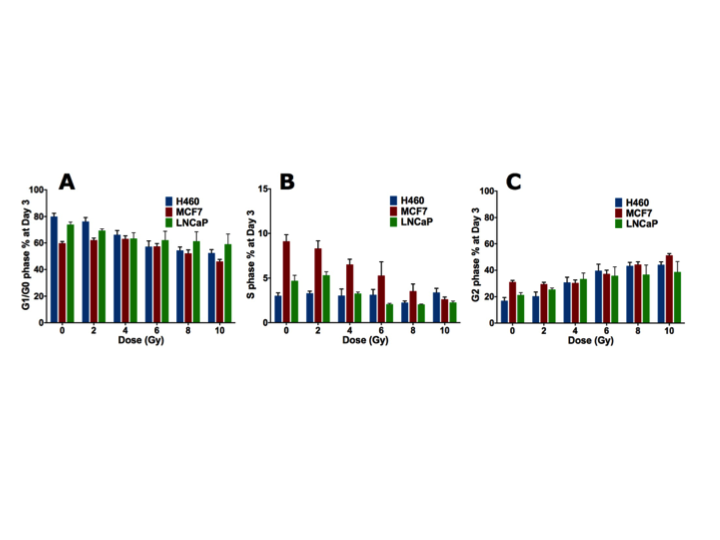

Supplement: S2 Fig — Relative fractions of (a) G1/G0-, (b) S-, and (c) G2-phase cells were counted in triplicate at 3-days post-irradiation via propidium iodide flow cytometry. Values are the mean ±SE from 3 independent experiments. (TIFF) [file pone.0135356.s002.tiff]

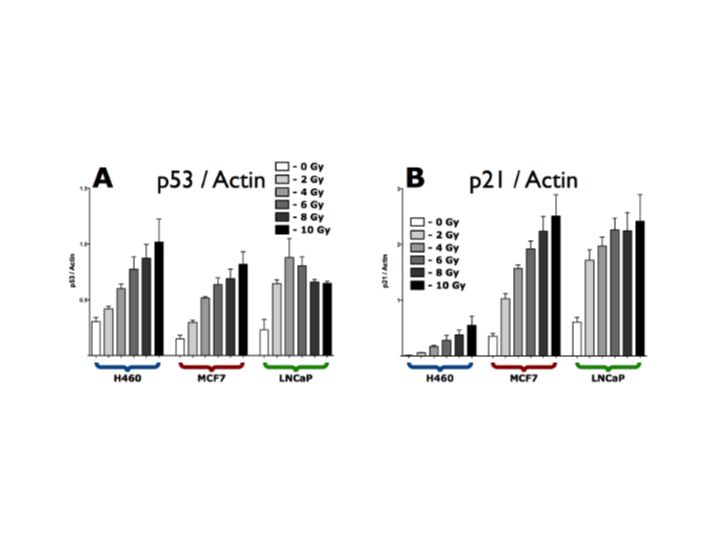

Supplement: S3 Fig — Whole cell lysates were prepared 3 days post-irradiation and Western blot analysis was conducted using anti–p53,–p21, and–actin antibodies. Results are the mean ±SE from two independent experiments. (TIFF) [file pone.0135356.s003.tiff]

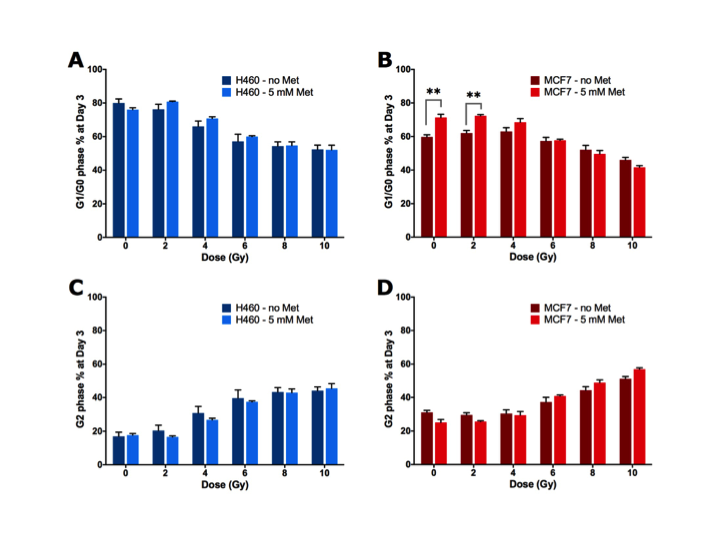

Supplement: S4 Fig — (a & b) G1/G0-phase fractions for (a) H460 and (b) MCF7 cells, and (c & d) G2-phase fractions for (c) H460 and (d) MCF7 cells were measured in triplicate at 3-days post-irradiation. Values are the mean ±SE from 3 independent experiments. ** p < 0.01 (unpaired two-tailed t-test). (TIFF) [file pone.0135356.s004.tiff]
